# Localized pancreatic Castleman disease presenting with extrahepatic dilatation of bile ducts: A case report and review of published cases

**DOI:** 10.1016/j.ijscr.2018.11.006

**Published:** 2018-11-20

**Authors:** Edson Gonçalves Ferreira Junior, Philippos Apolinario Costa, Larissa Melo Freire Golveia Silveira, Rafael Valois Vieira, Hugo Alessi Lima Martins Soares, Bruna Menon Loureiro, Nayane Carolina Pertile Salvioni, Jose Roberto Coelho Ferreira Rocha

**Affiliations:** Universidade Federal do Vale do São Francisco, Av. José de Sá Maniçoba, S/N – Centro, CEP: 56304-917, Petrolina, PE, Brazil

**Keywords:** Castleman disease, Case report, Pancreatic mass

## Abstract

Introduction: Castleman disease (CD) is a rare polyclonal lymphoproliferative disorder of unknown etiology, which usually develops in the mediastinum. It can also occur in the cervical, retroperitoneal and axillary regions. Localized pancreatic CD is quite rare [1].

Presentation of case: The authors herein present a case of a 34 years old female that was diagnosed during a symptomatic cholelithiasis evaluation. During the evaluation, an abdominal ultrasonography revealed a tumor at the head of the pancreas, which went on to generate a dilatation of the extrahepatic bile ducts. This finding was confirmed by abdominal magnetic resonance imaging (MRI). Subsequently, the patient underwent a laparotomy, where a capsulated tumor was found at the head of the pancreas with well-defined margins. The decision was made for tumor excision. The histopathology and immunohistochemistry established CD, hyaline vascular variation.

Discussion: The authors of the present paper also performed a literature review concerning Pancreatic CD, where there were found only 33 cases until the time of the writing of this paper, and we have subsequently carried out a retrospective analysis of all cases.

In a patient with atypical images, there might be a benefit from a preoperative diagnosis of CD, by using immunohistochemistry analysis in an image guided biopsy. Thus, avoiding unnecessary procedures and surgeries.

Conclusion: Localized pancreatic CD is a very rare condition with good prognosis, but it can mimic many common diseases, such as gastrointestinal stromal tumor (GIST), pancreatic neuroendocrine tumor or pancreatic adenocarcinoma.

## Highlights

• The authors present a unique case of localized pancreatic Castleman disease with extrahepatic bile duct dilatation.
• Pancreatic Castleman disease mimics gastrointestinal stromal tumor, pancreatic neuroendocrine tumor or adenocarcinoma.
• Preoperative diagnosis of pancreatic Castleman disease by image-guided biopsy and immunohistochemistry could improve outcome.

## 1 Introduction

CD, also known as angiofollicular or giant lymph node hyperplasia, is a rare lymphoproliferative disorder [[2], [3], [4]].

It was first described in 1954 as giant lymph node hyperplasia [5,6]. Initially, CD was reported as an indolent disorder, which was usually confined to a single lymph node group.

However, further case reports have gone on to describe a multicentric form of CD that often manifests a more malignant clinical course [2].

There has been no evidence found of any age predominance, as the condition affects children as well as adults, and neither any significant sex predilection has been found [7]. However, younger people are more likely to have the localized form [8]. Older adults and those with HIV infection are more likely to have the multicentric form [9].

Pancreatic localization of CD is very uncommon, with only a few reports in the literature. A list of the most common diagnoses of mass in the pancreas include adenocarcinoma, cystic tumors, and functioning or non-functioning neuroendocrine tumors.

Our objective is to present another case of pancreatic CD that mimics a pancreatic malignant neoplasm and to make a review of the topic with a retrospective analysis of all 33 cases published until now, to the best of our knowledge. This work was reported in line with the SCARE criteria [10].

## 2 Presentation of case

A 34 years old female presented herself to our service with a 3-year intermittent abdominal pain, associated with postprandial nausea and vomit. There was no history of fever, night sweats, decreased appetite, weight loss or bowel habit alterations. Family history was non-contributory. The physical examination was unremarkable.

Due to the possibility of cholelithiasis, an abdominal ultrasonography (US) was ordered. Abdominal US revealed a retroperitoneal tumor at the head of pancreas, biliary tract dilatation and cholelithiasis.

**Fig. 1:**
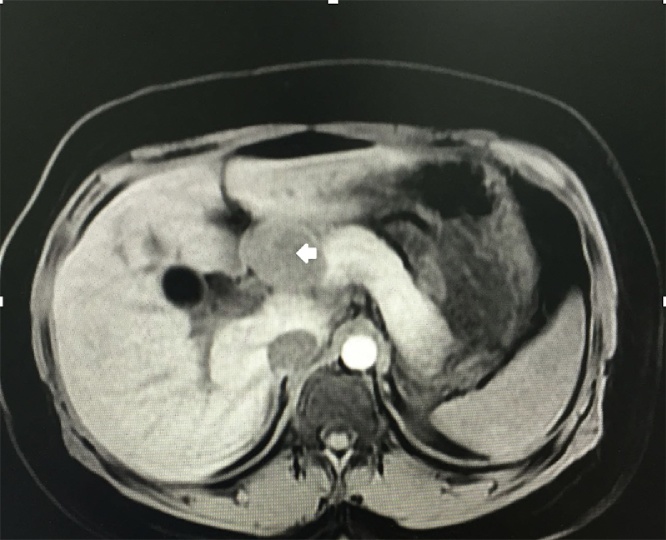
MRI axial T1 without contrast. The arrow points to the isointense 4 cm marginated mass in the pancreas head.

**Fig. 2:**
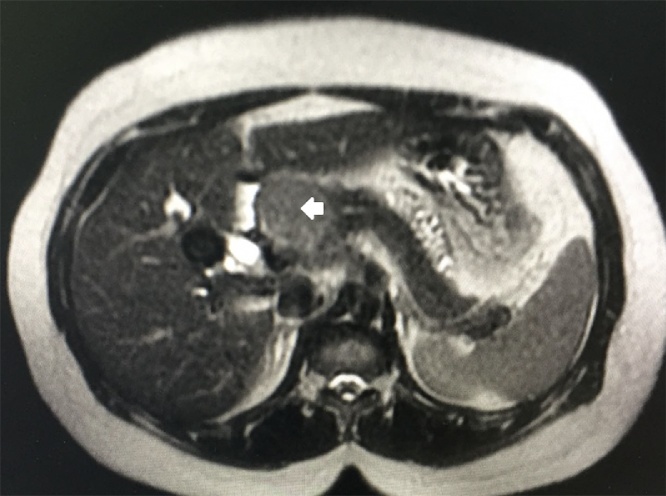
MRI axial T2. The arrow points to the mass with a signal intensity that is similar to the nomal pancreatic tissue.

**Fig. 3:**
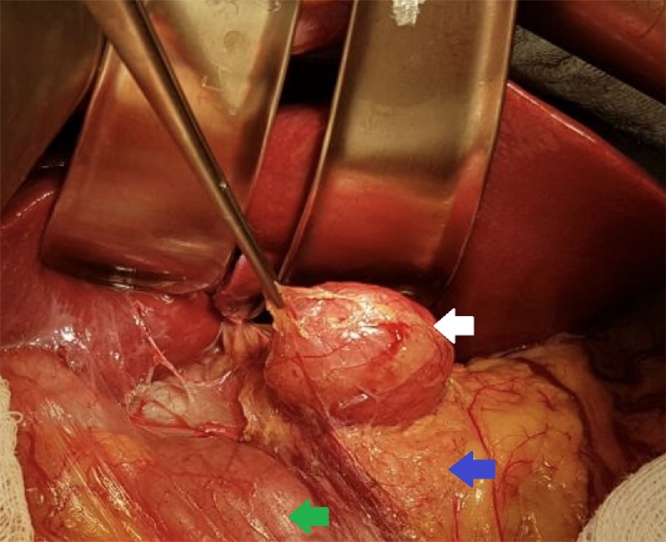
Intraoperative aspect. White arrow: Castleman tumor. Blue arrow: Pancreatic head. Green Arrow: Duodenum.

An abdominal magnetic resonance was ordered and showed an isointense smoothly marginated 4 cm mass in T1 (Fig. 1) and a signal intensity similar to the normal in T2 (Fig. 2). Homogenous enhancement, similar to the pancreas in T1 with contrast (Fig. 3). Cholelithiasis and common bile duct dilatation without Wirsung duct disturbance.

Based on the unspecific radiological findings and epidemiology, a provisional diagnosis of pancreatic adenocarcinoma, gastrointestinal stromal tumor (GIST) or pancreatic neuroendocrine tumor was made.

Adenocarcinoma was not the likely cause, as no consumptive symptoms were reported, and usually a pancreatic adenocarcinoma in T1 window with contrast as a hypointense mass rather than a normal pancreatic parenchyma.

Neither GIST showed signs of a strong diagnosis. Since it would appear as low signal intensity solid component in T1 and high signal intensity solid component in T2.

Pancreatic neuroendocrine tumor is usually hypointense relative to the pancreas in T1 and hyperintense relative to the pancreas in T2, but there is a range of signal intensities. Therefore, a pancreatic neuroendocrine tumor became our principal hypothesis.

The fact that the patient had a head pancreatic tumor with bile duct dilatation, associated with symptomatic cholelithiasis led to our preference for a surgical resection. Thus, a pancreatoduodenectomy was performed.

On exploration, we found an encapsulated mass bulging in the retroperitoneum at the pancreatic head (Fig. 3). There was also found a gallbladder with cholelithiasis, however, the liver, stomach and other organs appeared to be normal.

**Fig. 4:**
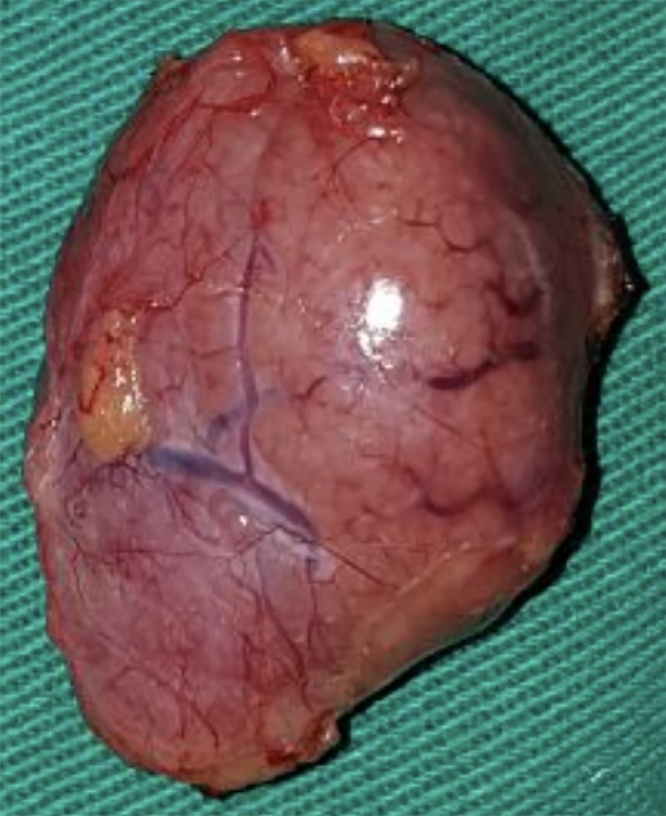
Macroscopic appearance. The tumor had smooth borders and was capsulated.

Based on the macroscopic aspect of the tumor, we opted for excision. The mass was completely excised, while preserving its capsule (Fig. 4), a feature that would not be typical in an pancreatic malignancy. Cholecystectomy was also performed without complications. No surgical approach in the bile duct or endoscopic retrograde cholangiopancreatography was made, since we understood that the dilation was due to extrinsic compression. The patient was discharged during the first postoperative day.

An anatomopathological examination showed chronic inflammation in the gallbladder and lymphoid proliferation in the excised tumor. Without immunohistochemistry, the lymphoid proliferation main possible diagnosis was a low grade B cells lymphoma or CD.

**Fig. 5:**
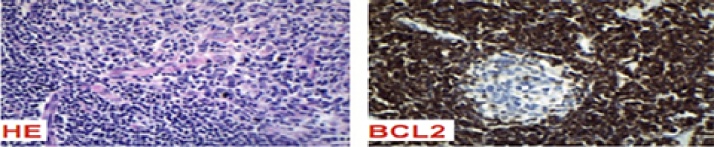
Immunohistochemistry. On the left: Prominent vascular proliferation and hyalinization of the vessel walls. On the right: Negative BCL2.

Immunohistochemistry demonstrated a prominent vascular proliferation and hyalinization of the vessel walls with an onion skin appearance and normal B and T lymphocytes distribution. CD20, CD3, CD10 and KI 67 were positive. BCL1 and BCL2 were negative (Fig. 5). Thus, we concluded that the diagnosis was CD, hyaline vascular variation.

The patient was seen on follow-up one month after the surgery. At that time, the patient was asymptomatic. The patient remained asymptomatic during all subsequent follow-ups.

## 3 Discussion

Little is known about the cause of this disorder of unknown origin [11]. The etiology and consequently the physiopathology of CD is not clearly known and actually most theories point to different etiological factors depending on the form that the disease presents, either the localized, unicentric or the multicentric form [12].

Seventy percent of cases present in the mediastinum and 20% occur in the axillary, cervical, inguinal and vulvar regions, while approximately 12% occur in the abdomen; mostly located in the pelvis, mesentery and perinephric regions [10]. Extrathoracic localizations are however reported with increasing frequency [2].

In the beginning, CD was classified histologically as two histopathological variants: hyaline vascular, (characterized by small hyaline-vascular follicles and interfollicular capillary proliferation), and plasma cell, (which is characterized by large follicles with intervening sheets of plasma cells) [[13], [14], [15]].

A mixed type of plasma cell and hyaline vascular type was further reported [7], however, rarely seen.

Clinically it can be classified as two types: localized and multicentric. The localized form is defined as a single, benign lesion, usually affecting young people [2].

Patients with multicentric disease, either hyaline‐vascular or plasma cell type, do not benefit from surgical management and should be candidates for multimodality therapy [16].

We only found one case of elevation of tumor markers in pancreatic CD. These results changed to normal 7 days after a pancreatic head mass excision. A possible explanation given by the author for the rise and fall of CA 19.9 could be intermittent compression of pancreatic ducts by the mass [17]. However, we found only one case that had a documented dilatation of pancreatic duct, and it did not present with a tumor marker elevation [18].

The current case to the best of our knowledge is the only case that demonstrates a biliary duct dilatation in a unicentric CD, documented by an MRI in a hyaline vascularh type, and no signs of tumor marker elevation either.

Imaging techniques like ultrasonography, computed tomography (CT) and MRI have been proven to be helpful in diagnosing retroperitoneal tumors. However, they show conflicts in their findings concerning CD, this incongruence is probably due to multiples histological types.

Therefore, imaging procedures should be considered for the differential diagnosis of a pancreatic mass [2]. However, the definitive diagnosis was based on the postoperative pathological findings, including CD located in the pancreas that was only confirmed after pathologic study of the surgical specimen [12].

The CD histological diagnostic is based on cell architecture, and therefore requires the study of the entire surgical specimen [15].

The adhesion of the tumor to the surrounding tissue and hypervascularity in the mass are characteristic features of the hyaline vascular type [19,20].

Immunohistochemical stains for ĸ and λ chains, L26, and UCHL-l [11]. Are currently able to define the diagnoses differentiating between low grade B cells lymphoma.

Multiple authors all have failed to establish CD as a diagnosis by endosonography controlled fine-needle aspiration biopsy (EUS-FNA) [8,[21], [22], [23]]. In none of these cases, cytological examination of material obtained from the tumor by EUS-FNA suggested adenocarcinoma, when in fact it was a mixed form of multicentric CD [8].

However, one case was successfully reported as a pancreatic CD preoperative diagnosis by EUS-FNA. The authors used flow cytometric analysis and a determined diagnosis. They claimed that occasional morphologic features on cytologic smears and on cell block section made the preoperative diagnoses possible [24].

The collaborators in Rhee et al in 2008 used an endoscopic ultrasonography guided trucut biopsy, but they could not establish a CD diagnosis. They were unable to make a differentiation from a low grade B-cell lymphoma [25], based only on the anatomopathological. Therefore, the association of the immunohistochemistry with trucut biopsy may be useful as preoperative diagnosis option.

Although there are no randomized studies, most published series agree that surgical complete resection is the best therapeutic option for the localized, unicentric form of CD including the plasma cell variant and mixed, with favorable long-term prognosis reports and no cases of malignant transformation [Table 1].

**Table 1:** XXX.

AUTOR	YEAR	COUNTRY	SEX	AGE	SYMPTOMS	TOPOGRAPHY	PANCREATIC DUCT DILATATION	BILE DUCT DILATATION	SURGERY	RECURRENCE	TIPE
LEPKE [26]	1982	USA	WOMAN	71	INCIDENTAL	BODY	NO	NO	WHIPPLE	DEAD	HV
LE VAN [27]	1989	EUA	WOMAN	64	INCIDENTAL	TAIL	NO	NO	DISTAL	NO FOLOW UP	HV
BROUSSAD [4]	1992	FRANCE	?	52	FEVER, WAIGHT LOSS	BODY	NO	NO	?	NO FOLOW UP	HV
INOUE [28]	1992	JAPAN	WOMAN	50	INCIDENTAL	HEAD	NO	NO	EXCISION	NO FOLOW UP	HV
CHAULIN [29]	1993	FRANCE	WOMAN	50	FEVER, FATIGUE, WAIGHT LOSS	BODY AND TAIL	NO	NO	DISTAL	NO FOLOW UP	MIXED
BAIKOVAS [17]	1994	AUSTRALIA	WOMAN	36	RIGHT ILIAC FOSSA PAIN	PERI	NO	NO	EXCISION	NO FOLOW UP	HV
LE BORGNE [30]	1999	FRANCE	MAN	54	FATIGUE, WEIGHT LOSS AND VAGE ABDOMINAL PAIN	HEAD	NO	NO	HEAD	11 MONTHS	PLASMA
KIM [31]	2001	KOREA	?	?	?	HEAD	?	?	EXCISION	NO FOLOW UP	PLASMA
CAMPRA [11]	2002	ITALY	WOMAN	27	EPIGASTRIC PAIN, ASTHENIA	HEAD	NO	NO	EXCISION	36 MONTHS	PLASMA
SOLER [32]	2003	SPAIN	MAN	36	INCIDENTAL	TAIL	NO	NO	DISTAL PANCREATECTOMY	1 YEAR	PLASMA
YILMAZ [2]	2004	TURKEY	WOMAN	56	FATIGUE, WEIGHT LOSS AND VAGE ABDOMINAL PAIN	BODY	NO	NO	WHIPPLE	3 MONTHS	HV
ERKAN [3]	2004	TURKEY	WOMAN	45	EPIGASTRIC PAIN	PERI	NO	NO	EXCISION	1 YEAR	PLASMA
GOETZE [21]	2005	GERMANY	MAN	53	INCIDENTAL	TAIL	NO	NO	DISTAL PANCREATECTOMY	2 YEARS	HV
SU [33]	2005	TAIWAN	WOMAN	38	abdominal fullness	NEEK	NO	NO	EXCISION	2 YEARS	HV
WASIELICA-BERGER [8]	2007	POLAND	MAN	54	GASTRIC FULLNESS, EPIGASTRIC PAIN, ADYNAMIA, ASTHENIA WEIGHT LOSS	MULTICENTRIC	NO	NO	EXCISED BIOPSIED	DEAD	MIXED
MAITHEL [34]	2007	USA	MAN	76	GASTRIC FULLNESS, JAUNDICE, ADYNAMIA, ASTHENIA WEIGHT LOSS	MULTICENTRIC	NO	YES	EXCISED BIOPSIED	6 MONTHS	PLASMA
MANGINI [35]	2007	ITALY	WOMAN	49	INCIDENTAL	BODY	NO	NO	EXCISION	NO FOLOW UP	HV
WANG [22]	2007	USA	MAN	58	INCIDENTAL	HEAD	NO	NO	WHIPPLE	NO FOLOW UP	HV
TUNRU-DINH [36]	2007	EUA	WOMAN	23	ABDOMINAL PAIN	TAIL	NO	N0	DISTAL PANCREATECTOMY	1 YEAR	HV
TALARICO [23]	2008	ROME	MAN	69	HIPOCONDRYAL PAIN AND FEVER	BODY	NO	NO	?	1 YEAR	MIXED
RHEE [25]	2008	JAPAN	WOMAN	50	INCIDENTAL	PERI	NO	NO	EXCISION	NO FOLOW UP	HV
CHARALABOPOULOS [1]	2010	GREEC	WOMAN	31	GASTRIC FULLNESS, EPIGASTRIC PAIN	PERI	NO	NO	DISTAL PANCREATECTOMY	2 YEARS	PLASMA
KHASHAB [24]	2011	USA	WOMAN	27	INCIDENTAL	BODY	NO	NO	EXCISION	NO FOLOW UP	HV
FU [9]	2012	INDIA	MAN	49	INCIDENTAL	TAIL	NO	NO	DISTAL PANCREATECTOMY	1O MONTH	HV
	2012	INDIA	MAN	39	ABDOMINAL PAIN	HEAD	NO	NO	EXCISION	NO FOLOW UP	HV
	2012	INDIA	MAN	74	INCIDENTAL	HEAD	NO	NO	EXCISION	26 MONTHS	PLASMA
APODACA-TORREZ [12]	2012	BRAZIL	MAN	64	ADYNAMIA, ASTHENIA WEIGHT LOSS	BODY	NO	NO	EXCISION	NO FOLOW UP	HV
CECKA [15]	2013	CZECH REPUBLIC	WOMAN	48	EPIGASTRICAL PAIN	TAIL	NO	NO	DISTAL PANCREATECTOMY LAPAROSCOPIC	1 YEAR	HV
MATSUMOTO [18]	2015	JAPAN	MAN	74	INCIDENTAL	HEAD	YES	NO	WHIPPLE	2 MONTHS	HV
ABDESSAYED [37]	2017	TUNISIA	WOMAN	34	ABDOMINAL PAIN	BODY	NO	NO	EXCISION	?	HV
CHENG [38]	2018	CHINA	WOMAN	48	INCIDENTAL	BODY	NO	NO	EXCISION	30 MONTHS	HV
	2018	CHINA	WOMAN	57	TIREDNESS AND FEVER	TAIL	NO	NO	EXCISION	?	HV
JAIN [39]	2012	INDIA	MAN	46	LEFT UPPER QUADRANT ABDOMINAL PAIN	TAIL	NO	NO	EXCISION	12 MONTH	HV
CURRENT CASE	2017	BRAZIL	WOMAN	34	ABDOMINAL PAIN	HEAD	NO	YES	EXCISION	1 YEAR	HV

Only one case of death during treatment and follow-up was reported in all localized pancreatic CD cases. However, this was attributed to comorbid, once the diagnoses were made in intraoperative in light of a different pathology, and the elderly patient died due to postoperative complications [26].

## 4 Conclusion

CD affecting the pancreas is a very rare occurrence, even nowadays with a great complementary arsenal for performing a diagnosis. Therefore, this is one of the reasons that CD is not usually included in the list of possible diagnosis. However, pancreatic CD should be taken into consideration in the differential diagnosis of a pancreatic mass.

These patients have a good prognosis in a majority of cases unlike malignant tumors. Accordingly, the possibility of a preoperative diagnosis in a patient with atypical findings in MRI or CT by image-guided biopsy associated with immunohistochemistry analysis would improve outcomes by avoiding useless tests, possible neoadjuvant chemotherapy, and finally would reduce surgical procedure morbidity. However, the definitive diagnosis will be performed based on the postoperative pathological findings.

## Conflicts of interest

None.

## Funding

Authors did not receive any funding for this work.

## Ethical approval

We do not require ethical approval to write a case report paper.

## Consent

Written informed consent was obtained from the patient for publication of this case report and accompanying images. A copy of the written consent is available for review by the Editor-in-Chief of this journal on request.

## Author contribution

1 Edson Gonçalves Ferreira Junior: Conceptualization, Methodology, Resources, Writing the paper, Writing – Review & Editing, Project Administration, Final approval
2 Philippos Apolinario Costa: Conceptualization, Methodology, Data collection, Data analysis/interpretation, Writing – Review & Editing, Final approval
3 Larissa de Melo Freire Gouveia Silveira: Conceptualization, Methodology, Data collection, Resources, Writing – Review & Editing, Final approval
4 Rafael Valois Vieira: Conceptualization, Methodology, Investigation, Writing – Review & Editing, Data collection, Resources, Final approval
5 Hugo Alessi L M Soares: Conceptualization, Methodology, Data collection, Writing – Review & Editing, Supervision, Final approval
6 Bruna Menon Loureiro: Conceptualization, Methodology, Data collection, Resources, Writing – Review & Editing, Final approval
7 Nayane Carolina Pertile Salvioni: Conceptualization, Methodology, Data collection, Resources, Writing – Review & Editing, Final approval
8 Jose Roberto Coelho Ferreira Rocha: Conceptualization, Methodology, Data collection, Resources, Writing – Review & Editing, Final approval

## Registration of research studies

Case reports don’t need to be registered.

## Guarantor

Edson Gonçalves Ferreira Junior.

## Provenance and peer reviewed

Not commissioned, externally peer reviewed.
